# Brigatinib and Alectinib for ALK Rearrangement-Positive Advanced Non-Small Cell Lung Cancer with or without Central Nervous System Metastasis: A Systematic Review and Network Meta-Analysis

**DOI:** 10.3390/cancers12040942

**Published:** 2020-04-10

**Authors:** Koichi Ando, Kaho Akimoto, Hiroki Sato, Ryo Manabe, Yasunari Kishino, Tetsuya Homma, Sojiro Kusumoto, Toshimitsu Yamaoka, Akihiko Tanaka, Tohru Ohmori, Hironori Sagara

**Affiliations:** 1 Division of Respiratory Medicine and Allergology, Department of Medicine, Showa University School of Medicine, 1-5-8 Hatanodai, Shinagawa-ku, Tokyo 142-8666, Japan; k_akimoto@med.showa-u.ac.jp (K.A.);; 2 Advanced Cancer Translational Research Institute (Formerly, Institute of Molecular Oncology), Showa University, 1-5-8 Hatanodai, Shinagawa-ku, Tokyo 142-8555, Japan

**Keywords:** alectinib, ALK rearrangement, brigatinib, central nervous system metastasis, indirect treatment comparison, NSCLC, progression-free survival, systematic review

## Abstract

To date, no head-to-head trials have compared the efficacy of brigatinib and alectinib against anaplastic lymphoma kinase (ALK) rearrangement-positive (ALK-p), ALK-inhibitor-naïve, advanced non-small cell lung cancer (NSCLC) with central nervous system (CNS) metastasis. We conducted an indirect treatment comparison (ITC) between brigatinib and alectinib, with crizotinib as a common comparator, using a Bayesian model with non-informative prior distribution and assessed the between-study heterogeneity of the studies. The primary efficacy endpoint was progression-free survival (PFS), and efficacy was ranked using the surface under the cumulative ranking (SUCRA) curve values. ITC analysis showed that there were no significant differences in PFS between the brigatinib and alectinib arms. However, the SUCRA values revealed that alectinib ranked the highest by efficacy in the overall patient population, whereas brigatinib ranked the highest by efficacy in the CNS metastasis sub-group. Although there were no significant differences in the incidence of G3–5 adverse events between the brigatinib and alectinib arms in the overall patient population, the data were deemed insufficient for the CNS metastasis sub-group analysis. This study provides critical information to clinicians regarding the efficacy of brigatinib for ALK-p, ALK-inhibitor-naïve, advanced NSCLC patients, with and without CNS metastasis. Larger randomized, controlled trials are warranted to confirm our results.

## 1. Introduction

**Figure 1:**
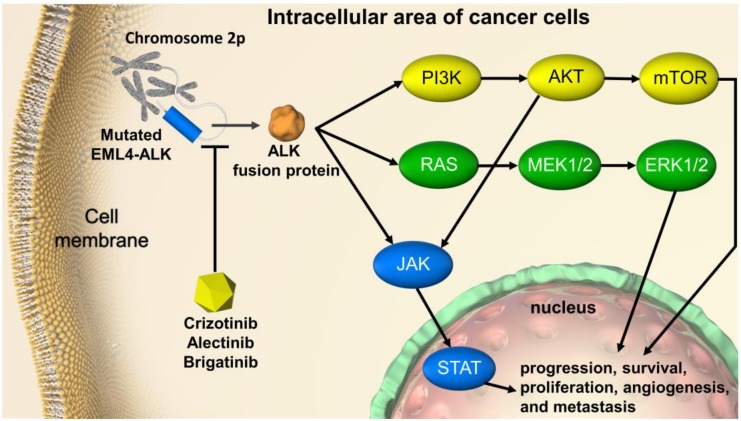
ALK-rearrangement-targeted cancer therapy in non-small cell lung cancer. EML4-ALK translocation activates PI3K-AKT, RAS, and JAK/STAT signaling cascades, thereby influencing tumor progression, survival, and growth. ALK inhibitors such as crizotinib, alectinib, and brigatinib act on mutated ALKs, such as ELM4-ALK, and suppress the production of ALK fusion protein resulting from ALK rearrangement. EML4-ALK, echinoderm microtubule-associated protein-like 4-anaplastic lymphoma kinase; ALK, anaplastic lymphoma kinase; PI3K, phosphatidylinositol-3 kinase; mTOR, mammalian target of rapamycin; RAS, reticular activating system; MEK, mitogen-activated extracellular signal regulated kinase; ERK, extracellular signal-regulated kinase (ERK); JAK, Janus kinase; STAT, signal transducer and activator of transcription.

Basic, clinical, and translational research conducted over the last 10 years has enhanced our understanding of the molecular disease mechanisms of non-small cell lung cancer (NSCLC). As a result, the treatment strategy for NSCLC patients has undergone a remarkable evolution [1,2]. Nevertheless, lung cancer remains the leading cause of cancer-related deaths worldwide. In 2018, lung cancer accounted for 13% of all cancer cases, with a 5-year relative survival rate of only 18% [3]. Only 16% of cases are diagnosed at a localized stage, at which the 5-year survival rate is 56%. Most lung cancers are diagnosed at an advanced stage, at which the 5-year survival rate is only 5% [3,4]. Lung cancer has been associated with a high rate of central nervous system (CNS) metastasis [5,6]. In particular, the frequency of CNS metastasis is found to be high in cases of anaplastic lymphoma kinase (ALK) rearrangement-positive (ALK-p) NSCLC, and it contributes to the deterioration of quality of life and prognosis [7,8,9]. Non-small cell lung cancer (NSCLC) accounts for more than 84% of all lung cancers [10]. With the extension of survival time due to the advances in lung cancer treatment, the treatment of ALK-p NSCLC with CNS metastasis is becoming increasingly important. However, therapeutic strategies for ALK-p NSCLC with CNS metastasis have not yet been developed [11]. Figure 1 illustrates the signaling cascade affected by ALK gene translocation. The most common gene translocation is of echinoderm microtubule-associated protein-like 4-ALK (EML4-ALK), which promotes ALK phosphorylation, thus activating phosphatidylinositol-3 kinase-AKT (PI3K-AKT), reticular activating system (RAS), and Janus kinase/signal transducer and activator of transcription (JAK/STAT) signaling cascades. This influences not only carcinogenic drivers and tumor growth, but also other organ metastases, including central nervous system metastases [12,13,14,15].

Crizotinib, an ALK and c-ros oncogene 1 (ROS1) inhibitor, is used to treat ALK-p, advanced, or recurrent NSCLC and has been approved in the United States of America as a treatment option for ROS1-positive metastatic NSCLC [16]. Alectinib, an ALK inhibitor, has a benzo[b]carbazole skeleton, unlike crizotinib or ceritinib, and has a high selectivity for ALK compared to other ALK inhibitors [17]. Alectinib has been reported to be effective in patients who have acquired resistance to crizotinib and who have experienced a relapse of distant metastases, including CNS metastases [18]. Two phase III studies (J-ALEX and ALEX) comparing alectinib and crizotinib for ALK-p, ALK-inhibitor-naïve, advanced NSCLC showed that alectinib significantly increased progression-free survival (PFS) compared to crizotinib [19,20]. The J-ALEX study showed that the incidence of Grade 3 or higher adverse events was less common with alectinib (32%) than with crizotinib (57%) [20]. The primary toxicities of crizotinib include visual impairment; gastrointestinal complications, such as diarrhea and nausea; and impaired liver function, while the primary toxicities of alectinib are taste disorders, myalgia, eruptions, and, as with other kinase inhibitors, interstitial pneumonia [19,20]. Based on these results, the National Comprehensive Cancer Network (NCCN) guidelines recommend alectinib as a first line treatment option [16].

In 2017, brigatinib was approved by the United States Food and Drug Administration (US FDA) as an ALK inhibitor only for patients who have progressed or are intolerant to the first-generation ALK TKI, crizotinib [21]. A phase III study comparing the efficacy and safety of brigatinib and alectinib in patients with recurrent cancers during crizotinib treatment is currently ongoing [22]. Regarding ALK-inhibitor-naïve cases, a Phase III study of ALK-p, ALK-inhibitor-naïve, advanced NSCLC has demonstrated that the PFS of brigatinib-treated patients was significantly longer than that of crizotinib-treated patients [23]. Brigatinib was reported to be relatively tolerable. The most common G3–5 adverse events observed for brigatinib treatment were an increases in creatine phosphokinase (24.3%), lipase (14.0%), and hypertension (11.8%) [23]. A noteworthy finding of this study was that brigatinib-induced PFS prolongation was observed both in cases with and without CNS metastasis, although ALK inhibitors generally have poor CNS permeability and are therefore reported to have diminished efficacy in CNS metastasis [23]. A phase II study has demonstrated the efficacy of brigatinib against CNS metastatic lesions [24]. Furthermore, it has also been demonstrated in an ALK-p NSCLC mouse model with CNS metastasis that the antitumor effects of low concentrations of brigatinib were equivalent to those of crizotinib [25]. Thus, brigatinib is a highly effective ALK inhibitor that can be used for the treatment of ALK-p NSCLC patients with CNS metastasis [25]. 

However, there are no reports of any large-scale, head-to-head, randomized, controlled trials (RCT) in ALK-inhibitor-naïve NSCLC patients with CNS metastasis that compare currently the recommended dosages and administration frequency of ALK inhibitors or brigatinib with those of alectinib, which is currently positioned as a first-line treatment. Although a phase III trial comparing the efficacy and safety of brigatinib and alectinib in NSCLC patients, showing progression or recurrence and undergoing crizotinib treatment, is ongoing [22], it is mainly for patients who have undergone crizotinib treatment and does not include any previously untreated or ALK naïve patients. A comparison of the efficacy and safety of brigatinib and alectinib for ALK-p, ALK-inhibitor-naïve NSCLC with CNS metastasis is extremely important to develop a treatment strategy for CNS metastasis in NSCLC [11]. We believe that a comparison of brigatinib and alectinib is critical, not only in patients with NSCLC with CNS metastasis, but also in all patients with ALK-p, ALK-inhibitor-naïve, advanced NSCLC. Hence, we have selected our study population to include patients with ALK-p, ALK-inhibitor-naïve, advanced NSCLC, with and without CNS metastasis. 

Although RCTs should be conducted to compare the efficacy and safety of drugs, such studies are time-consuming, expensive, and labor-intensive. In the absence of RCTs, an indirect treatment comparison (ITC) can be made through common comparisons by using the statistical method of network meta-analysis. ITCs may allow us to obtain useful information faster than RCTs [26]. We used a Bayesian model [27] to indirectly compare the efficacy and safety of brigatinib and alectinib for the treatment of ALK-p NSCLC, with and without CNS metastasis. The advantage of a network meta-analysis is that we can make indirect comparisons between drugs through a common comparison target, even in the absence of any existing direct comparison [28,29]. The purpose of this study was to compare the efficacy and safety of brigatinib and alectinib for ALK-p, ALK-inhibitor-naïve, advanced NSCLC, with and without CNS metastasis, by Bayesian network meta-analysis. 

## 2. Methods

### 2.1. Systematic Review

We conducted a comprehensive literature search to identify published reports of RCTs of brigatinib and alectinib from 1946 to the present. On 2 January 2020, four databases (PubMed, Cochrane Library, EMBASE, and SCOPUS) were searched. We used keywords such as NSCLC, ALK inhibitors and their Medical Subject Headings (MeSH) terms to build a search strategy. For example, the following strategy was used to search PubMed: (“Non-Small Cell Lung Cancer” OR “Non-Small Cell Lung Carcinoma” OR “Non Small Cell Lung Carcinoma” OR “Non-Small-Cell Lung Carcinoma” OR “Nonsmall Cell Lung Cancer” OR “Non-Small-Cell Lung Carcinomas” OR “NSCLC”) and (“Alectinib” OR “Alecensa” OR “RO5424802” OR “CH5424802” OR “Brigatinib” OR “AP26873”). In addition, we reviewed the reference lists of the retrieved studies to avoid the risk of missing relevant studies that met the inclusion criteria. If a database did not have enough information about the study, an email query was sent to the corresponding author. Based on this search strategy used in PubMed, we also searched EMBASE, CENTRAL, and SCOPUS. The primary aim of this systematic review (registration: UMIN-CTR no. UMIN000036256) of the literature was to identify all publicly available RCTs to support the comparison of efficacy and safety between brigatinib and alectinib in patients with ALK-p ALK inhibitor naïve advanced NSCLC with CNS metastasis.

We searched the literature for studies that met the inclusion criteria, in addition to the phase III trials that had already been identified [19,20,23]. To identify all relevant studies and minimize publication bias, we conducted a review of the references presented in the articles and a manual search of relevant articles. The study was performed according to the Preferred Reporting Items for Systematic Review and Meta-analysis (PRISMA) statement [30] and the PRISMA extension statement for network meta-analysis [31]. Two investigators (K.Ando and TY) independently conducted literature searches. Using the predefined Patients, Interventions, Comparison, Outcomes, and Study design (PICOS) approach, inclusion and exclusion criteria were adapted for the studies retrieved in this systematic review to address the clinical or methodological heterogeneity between studies and to ensure the validity of the indirect comparison analysis.

### 2.2. Quality Evaluation

The risk-of-bias (RoB) tool recommended by the Cochrane Collaboration was used to assess the qualities of RCTs included in the present analysis [32]. The following parameters were assessed as being high, unclear, or low: (1) random sequence generation (selection bias due to inadequate generation of a randomized sequence), (2) allocation concealment (selection bias due to inadequate concealment of allocations prior to assignment), (3) blinding of participants and personnel (performance bias due to knowledge of the allocated interventions by participants and personnel during the study), (4) blinding of the outcome assessment (detection bias due to knowledge of the allocated interventions by outcome assessors), (5) incomplete outcome data (attrition bias due to the amount, nature, or handling of incomplete outcome data), (6) selective reporting (reporting bias due to selective outcome reporting), and/or (7) other bias (bias due to problems not covered elsewhere). We assessed heterogeneity between studies using *I^2^* statistics [33]. *I^2^* statistical values range from 0% to 100%, with a higher value corresponding to higher heterogeneity. Statistical significance was assessed for the *I^2^* statistic, and *p* < 0.05 was considered to indicate statistically significant heterogeneity [33].

### 2.3. Inclusion and Exclusion Criteria (Predefined PICOS)

The purpose of this study was to compare the efficacy and safety of approved dosages of brigatinib and alectinib in adult ALK-naïve, advanced NSCLC patients. Therefore, we selected the following inclusion criteria for this study: a minimum age of 18 years; a histological or cytological confirmation of advanced or metastatic ALK-p NSCLC with at least one measurable lesion according to the Response Evaluation Criteria in Solid Tumors (RECIST), version 1.1,25, with an Eastern Cooperative Oncology Group (ECOG) performance status of 0 to 2 (on a five-point scale, with higher numbers reflecting greater disability); and no previous ALK-targeted therapy.

### 2.4. Interventions/Comparisons

Treatments eligible for this ITC analysis were oral brigatinib at a dose of 180 mg once daily after a 7-day lead-in period of 90 mg once daily and oral alectinib at a dose of 600 or 300 mg twice daily, all of which are licensed or recommended dosages and modes of administration. Studies with either of these as a treatment group were included in our analysis. It was assumed that crizotinib was the common comparator for both therapeutic agents. This is because, prior to the approval of alectinib and brigatinib, crizotinib was the first choice in the initial treatment for ALK-p, untreated NSCLC and is considered an appropriate, comparative target in RCTs.

### 2.5. Outcomes

The primary efficacy endpoint for our analysis was PFS, one of the most common efficacy endpoints in the field of clinical oncology, which is expressed in terms of the hazard ratio (HR) and 95% credible intervals (CrIs). The primary safety endpoint was the incidence of “any adverse events” of grades 3–5 (G3–5AAEs), which is expressed in terms of the odds ratio (OR) and 95% credible intervals (CrIs). To rank the efficacy of both treatments, the values of the surface under the cumulative ranking (SUCRA) curves for PFS were also calculated [34]. Analysis for all participants and subgroup analysis for patients with CNS-metastasis were performed. These predefined endpoints were analyzed only if data were available from the included studies. Two authors (K.Ando and TY) extracted relevant data independently, and a third author (TO) was consulted to resolve discrepancies when necessary.

### 2.6. Study Design

The studies that were eligible for inclusion in the present ITC were defined as phase III studies in double-blind, parallel group RCTs. At least one preset efficacy or safety endpoint should have also been available in the study for ITC analysis.

### 2.7. Statistical Analysis Method of Indirect Comparison

ITCs of brigatinib and alectinib for the predefined safety and efficacy endpoints were performed using the Bayesian network meta-analysis method in accordance with the established methodology outlined by the National Institute for Health and Care [35,36,37]. This statistical method is a well-established statistical method of ITC [38,39,40] and is supported not only by the International Society for Pharmacoeconomics and Outcome Research (ISPOR) guidelines for indirect comparison and network meta-analysis, but also by the National Institute for Health and Clinical Excellence and Haute Autorité de Santé [41,42]. In the absence of RCTs demonstrating direct comparisons, the methodology of indirect comparison is useful for comparing treatment regimens [26] and has also been used in several other fields [43,44,45,46]. For our analysis, we used the standard method of ITC, as described by Dias et al. [35,36,37].

This analysis adopted the Bayesian model, which assumes heterogeneity between the included studies [36]. This Bayesian analysis involved the use of a noninformative prior distribution and Gibbs sampling using the Markov chain Monte Carlo method to estimate the posterior distribution of treatment effects.

Iteration was performed 50,000 times, with the first 10,000 iterations considered to be burn-in samples. The Brooks-Gelman-Rubin (BGR) diagnostic method was used to assess model convergence [47,48]. The treatment effect was expressed in terms of the HR and OR with 95% CrIs. We derived 95% CrIs at 2.5% and 97.5% of the posterior distribution. Results were interpreted as being not significant if the 95% CrI exceeded the ineffective line (i.e., the HR or OR was 1). Analyses were performed for all participants and for the limited population of NSCLC patients with CNS metastasis as a subgroup.

Network meta-analysis facilitates both the comparison and ranking of treatment groups. In the present ITC, the treatments were ranked based on the SUCRA values calculated from the Bayesian analysis [34]. SUCRA values ranged from 0% to 100%, with higher values indicating that the treatment was relatively more effective; a value of 100% indicated that the drug was the most ideal treatment [34]. The analysis was performed using OpenBUGS 1.4.0 (MRC Biostatistics Unit, Cambridge Public Health Research Institute, Cambridge, UK), and STATA (ver. 14, StataCorp, College Station, TX, USA) was used to create graphics for the presentation of the results.

### 2.8. Ethical Aspects

Institutional review board approval and patient consent were waived due to the nature of the review performed in this study.

## 3. Results

### 3.1. Systematic Review

**Figure 2:**
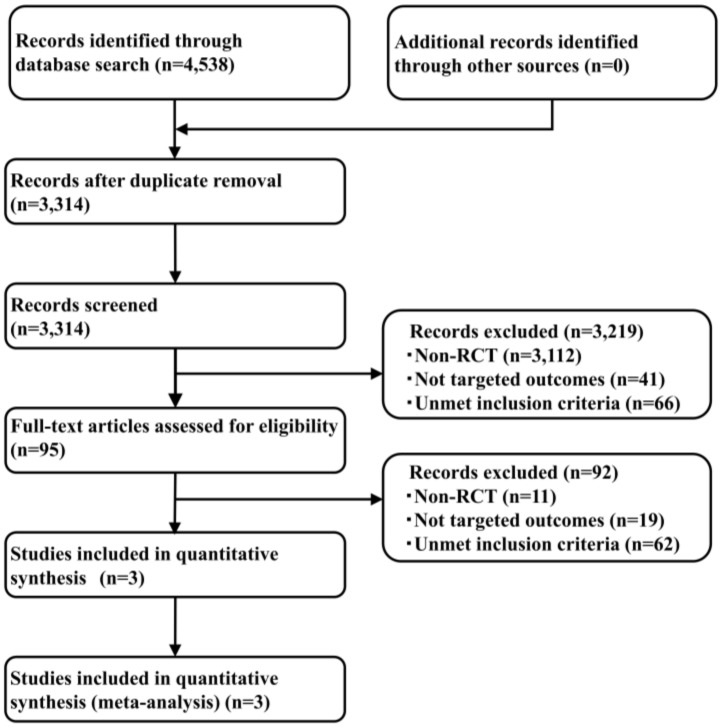
A flow diagram of the study selection process.

By performing a systematic literature review, studies were identified (414 from PubMed, 1353 from EMBASE, 155 from CENTRAL, and 2616 from SCOPUS), and 3314 articles were retained after the removal of duplicates. The adoption of the PICOS approach led to the retention of two studies, one of which compared brigatinib with crizotinib (ALTA-1L) [23], and the other two compared alectinib and crizotinib (ALEX and J-ALEX) [19,20] for the ITC analysis. The results of the systematic review did not find any new enrollment studies other than the three previously identified studies. The study selection process is shown in Figure 2, key inclusion criteria are shown in Table 1, and the primary characteristics of the included studies are shown in Table 2. Analysis was performed for all participants comprising 785 patients (275 from ALTA-1L, 303 from ALEX, and 207 from J-ALEX).

The common comparative group in both the brigatinib and alectinib studies was crizotinib. Although study data were available for an ITC analysis of the predefined primary efficacy endpoint (PFS), the data reported in the CNS metastasis subgroup for the licensed doses of brigatinib and alectinib were not sufficient for the ITC analysis of the predefined safety endpoints (G3–5AAEs). Hence, the ITC analysis of the primary safety endpoints was performed for all participants. The preferred model convergence was confirmed in all of the analyses using the BGR diagnostic method.

### 3.2. Primary Efficacy Endpoint: Progression-Free Survival

**Figure 3:**
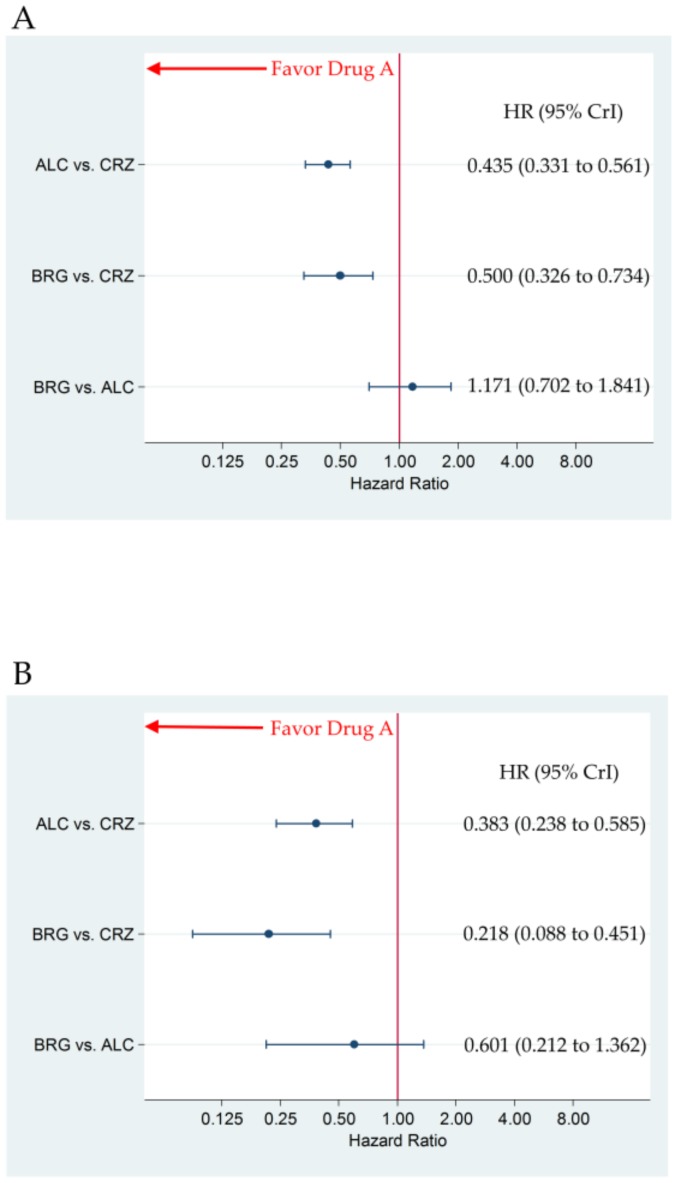
The comparative efficacy, in terms of progression-free survival, of brigatinib 180 mg once daily (7-day run-in period of 90 mg once daily) and alectinib 300/600 mg twice daily. The comparisons are expressed as drug A versus drug B. Data are expressed in terms of hazard ratio (HR) and 95% credible intervals (CrIs). (**A**) Comparison in all participants; (**B**) Comparison in the patient group with central nervous system metastasis. ALC, alectinib; CRZ, crizotinib; BRG, brigatinib.

**Figure 4:**
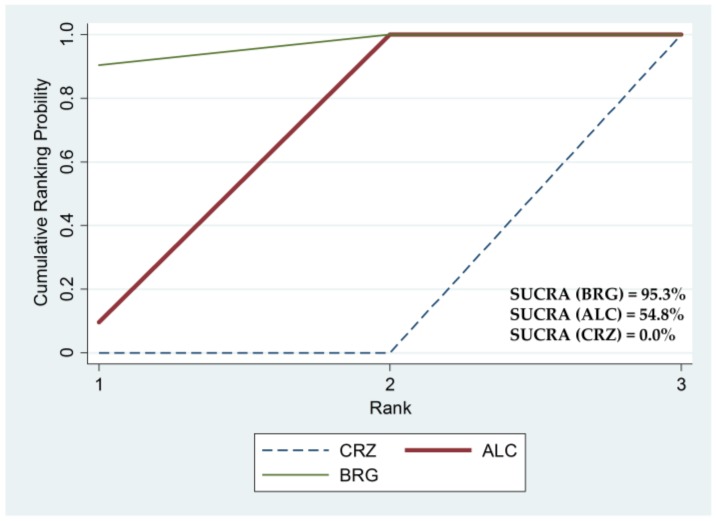
Surface under the cumulative ranking (SUCRA) curve for non-small cell lung cancer (NSCLC) with central nervous system (CNS) metastasis. SUCRA values are expressed as percentages of the areas under the curve, with higher SUCRA values indicating better treatment. BRG, brigatinib; ALC, alectinib; CRZ, crizotinib.

There were no significant differences in PFS between brigatinib and alectinib, with HR (brigatinib versus alectinib) (95% CrI) of 1.171 (0.702 to 1.841) for all participants and 0.601 (0.212 to 1.362) for the subgroup with CNS metastasis, respectively. Brigatinib and alectinib improved PFS compared to crizotinib, with HR (95% CrI) of 0.500 (0.326 to 0.734) and 0.435 (0.331 to 0.561) for all participants, and 0.218 (0.088 to 0.451) and 0.383 (0.238 to 0.585) for the subgroup with CNS metastasis, respectively (Figure 3A,B). The SUCRA values for PFS with brigatinib, alectinib, and crizotinib were 65.2%, 84.9%, and 0.0% for all participants, and 95.3%, 54.8%, and 0.0% for the subgroup with CNS metastasis, respectively (Figure 4).

### 3.3. Incidence of G3–5AAEs

**Figure 5:**
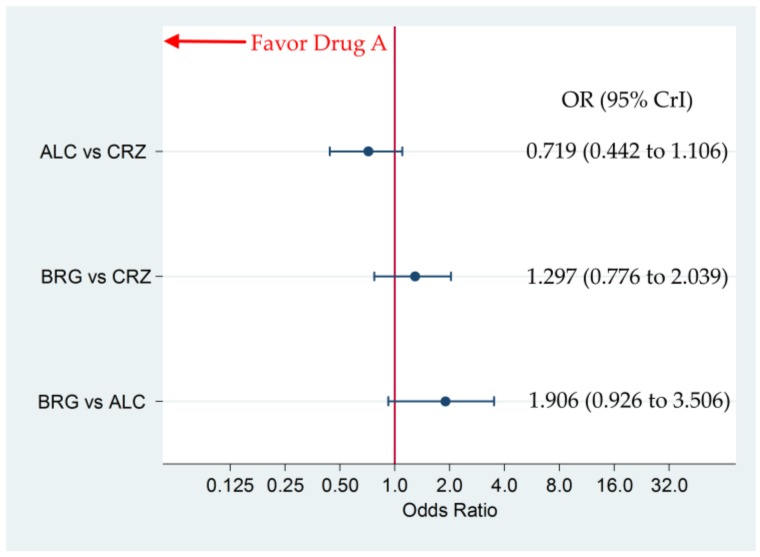
The comparative safety, in terms of “any adverse events” of grades 3–5 (G3–5AAEs), of brigatinib 180 mg once daily (7-day run-in period of 90 mg once daily) and alectinib 300/600 mg twice daily in all participants. The comparisons are expressed as drug A versus drug B. Data are expressed as odds ratio (OR) and 95% credible intervals (CrIs); ALC, alectinib; CRZ, crizotinib; BRG, brigatinib.

**Figure 6:**
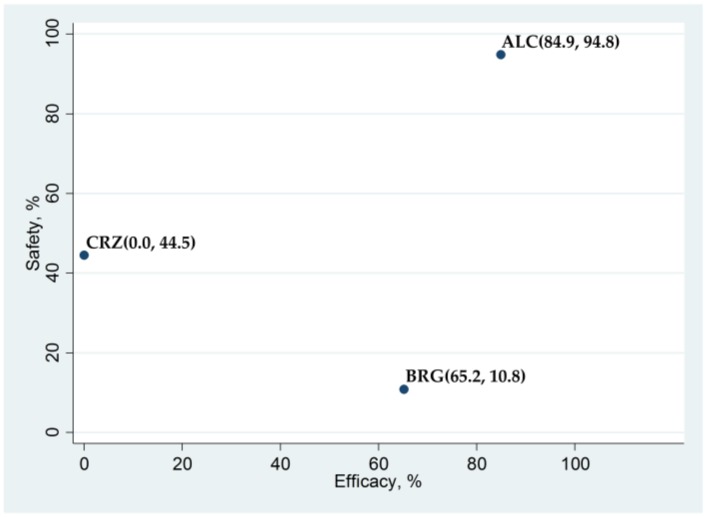
A scatter plot of the surface under the cumulative ranking (SUCRA) values of the efficacy and safety of three anaplastic lymphoma kinase (ALK) inhibitors in terms of progression-free survival (efficacy) and G3–5AAEs (safety) for the overall patient population. Data are presented as (SUCRA in PFS, SUCRA in G3–5AAEs) for each ALK inhibitor. ALC, alectinib; CRZ, crizotinib; BRG, brigatinib; G3–5AAEs, “any adverse events” of grades 3–5.

For all participants, there were no significant differences in G3–5AAEs between brigatinib and alectinib, brigatinib and crizotinib, and alectinib and crizotinib, with OR (95% CrI) of 1.906 (0.926 to 3.506), 1.297 (0.776 to 2.039), and 0.719 (0.442 to 1.106), respectively (Figure 5). The SUCRA values for G3–5AAEs of brigatinib, alectinib, and crizotinib were 10.8%, 94.8%, and 44.5% for all participants, respectively (Figure 6).

Figure 6 shows a scatter diagram of the SUCRA of the efficacy and safety outcomes of the three ALK inhibitors for the overall patient population. In terms of efficacy, alectinib (SUCRA = 84.9%) ranked the highest, brigatinib (SUCRA = 65.2%) was second, and crizotinib ranked third (SUCRA = 0.0%). However, alectinib ranked the highest in terms of safety (SUCRA = 94.8%), followed by crizotinib (SUCRA = 44.5%), and finally, brigatinib (SUCRA = 10.8%).

### 3.4. Bias Assessment

**Figure 7:**
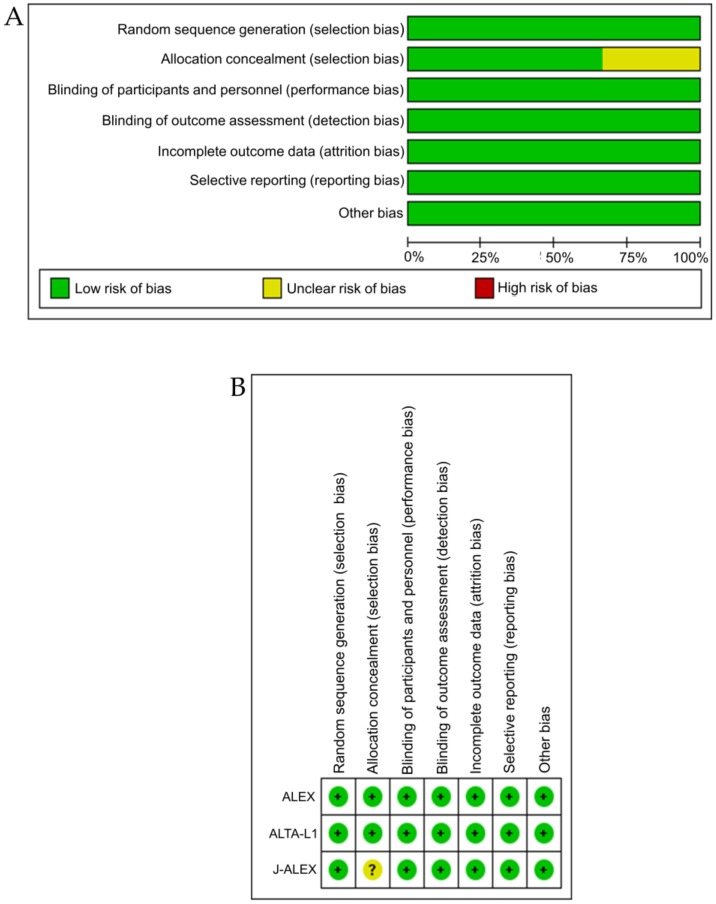
A risk-of-bias graph and summary. (**A**) A risk-of-bias graph: a review of authors’ judgments regarding each risk-of-bias item, presented as percentages across all included studies. (**B**) A risk-of-bias summary: a review of authors’ judgments regarding each risk-of-bias item for each included study.

The Cochrane risk-of-bias tool (RoB) revealed a low risk-of-bias for all studies included in this analysis. A risk-of-bias graph and summary are presented in Figure 7A,B. The bias risk was generally low, except for one article in which the selection bias was reported as an unclear risk.

In this study, we adopted the risk of bias (RoB) tool published by Cochrane in 2008 as a method of bias assessment for study inclusion. According to the PRISMA guidelines, RoB is widely recognized as a method of evaluating the risk of bias and is used even in current systematic reviews; RoB2 is the latest version of this tool. In this study, we evaluated the risk of bias of study inclusion not only by using RoB, but also by using RoB2, which has six domains: the risk of bias arising from the randomization process, the risk of bias due to deviations from the intended interventions, missing outcome data, the risk of bias in the measurement of the outcome, the risk of bias in the selection of the reported result, and the overall risk of bias. The authors assessed the bias risk of three included studies (ALEX, J-ALEX, and ALTA-1L) and found that the risk of bias due to deviations from the intended interventions in one study (J-ALEX) was unclear. Other than that, all were judged as low risk.

Both ALEX and J-ALEX compared alectinib and crizotinib. Therefore, the between-study heterogeneity was evaluated in these two trials. The results showed that I^2^ had a value of 23% (*p* = 0.25) for all patients and 0% (*p* = 0.32) for the subgroup with CNS metastasis, indicating no statistically significant difference in between-study heterogeneity.

### 3.5. Comparison with Analysis Using Another Statistical Method

In this study, we performed statistical analysis by using the Bayesian approach. To confirm the results obtained in this analysis, the frequentist approach was adopted by using STATA (ver. 14, StataCorp, College Station, TX, USA) [28]. We compared the “mean ranks” of alectinib, brigatinib, and crizotinib using these two statistical methodologies. The “mean rank” indicates the expected rank of each drug. The mean rank of PFS in cases with CNS metastasis was 1.905, 1.095, and 3.00 for alectinib, brigatinib, and crizotinib, respectively, according to the Bayesian approach (using OpenBUGS statistical software). The “mean ranks” calculated by the frequentist approach (using STATA statistical software) were 1.900, 1.100, and 3.00 for alectinib, brigatinib, and crizotinib, respectively. Both showed substantial agreement. Similarly, the mean ranks of PFS calculated by the Bayesian approach for alectinib, brigatinib, and crizotinib in all participants were 1.303, 1.697 and 3.00, respectively. The mean ranks of PFS calculated by the frequentist approach for alectinib, brigatinib, and crizotinib in all participants were 1.300, 1.700 and 3.00, respectively. The analysis of the overall population also yielded similar “mean ranks” between the two statistical methodologies. Thus, we confirmed the validity of our results and conclusions using two statistical methods.

### 3.6. Sensitivity Analysis

For ALEX, the dose of alectinib was 600 mg, and for J-ALEX, the dose was 300 mg. ALEX and ALTA-L1 are international collaborative trials, while J-ALEX is a trial for Japanese patients. To determine whether the inclusion of J-ALEX in this analysis might have changed the conclusion, we conducted sensitivity analysis by including only ALEX and ALTA-L1 in our analysis. As a result, in a sub-analysis of CNS metastasis cases, although there was no significant difference between brigatinib and alectinib, with HR (95% CrI) of 0.561 (0.195 to 1.284), SUCRA was higher for brigatinib (SUCRA = 96.3) compared to for alectinib (SUCRA = 53.8). Thus, the results of the sensitivity analysis revealed that the exclusion of J-ALEX did not affect the conclusion of the meta-analysis.

## 4. Discussion

The present ITC analysis aimed to compare the efficacy and safety of brigatinib and alectinib in patients with ALK-p, advanced NSCLC, with and without CNS metastasis. The results show that there was no significant difference in terms of PFS between brigatinib and alectinib for all of the participants, including those with ALK-p NSCLC and ALK-p advanced NSCLC with CNS metastasis. Efficacy ranking analysis for the three ALK inhibitors—brigatinib, alectinib, and crizotinib—revealed that alectinib ranked the highest in the overall ALK-p NSCLC patient population, whereas brigatinib ranked the highest in patients with ALK-p advanced NSCLC with CNS metastasis. There was no significant difference in the incidence of G3–5AAEs between brigatinib and alectinib in the overall patient population with ALK-p NSCLC.

We used Cochran’s proposed RoB and RoB2 to assess the risk of bias in the included studies. As a result, J-ALEX was assessed to have an "unclear risk", but the bias risk was generally low. In addition, no statistically significant heterogeneity was detected between ALEX and J-ALEX. The sensitivity analysis revealed that the inclusion of J-ALEX in the ITC did not affect the overall results. Thus, all these analyses confirm the reliability of the results of the ITC.

Meta-analyses of single-arm clinical trials with different doses of ALK inhibitors, such as alectinib, ceritinib, brigatinib, and crizotinib, have shown that the combined objective response rates for ALK-p NSCLC with CNS metastases were 79%, 45%, 48%, and 18%, respectively [49]. However, the patients enrolled in this meta-analysis included those previously treated with ALK inhibitors at various dosages and administration durations, beyond what is approved. Hence, in this indirect comparison, we compared, for the first time—using PFS as an efficacy endpoint—brigatinib, alectinib, and crizotinib, with the recommended or approved dosages and modes of administration for advanced NSCLC patients and showed that alectinib ranked the highest for the overall patient population with ALK-p NSCLC, whereas brigatinib ranked the highest for ALK-p NSCLC with CNS metastasis patients. ALTA-1L reported that brigatinib was more effective than crizotinib, especially with respect to PFS, in cases with CNS metastasis. Our findings not only confirmed those of ALTA-1L, but also showed, for the first time, that brigatinib had greater (albeit, not significantly greater) efficacy than alectinib in patients with CNS metastasis.

Previous reports have compared ALK inhibitors by using network meta-analysis [50,51]. However, these studies included non-approved dosages and administration frequencies. Moreover, the efficacy of brigatinib had not been evaluated in the subset of CNS metastasis cases. In one report, brigatinib was not included in the treatment arm [51]. Thus, our study was the first to assess the efficacy of brigatinib versus alectinib at the approved dosages and administration frequencies for ALK-naïve, advanced NSCLC with CNS metastasis and to show that brigatinib was more effective than alectinib for such cases.

**Figure 8:**
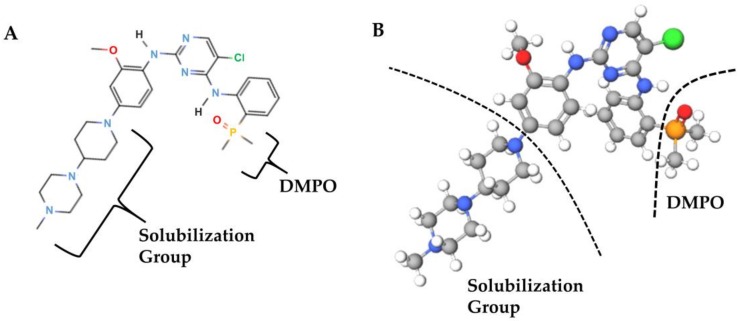
Brigatinib (**A**) and a ball-and-stick representation of brigatinib (**B**). Brigatinib has a dimethylphosphine oxide (DMPO) group, a feature not found in other ALK inhibitors. The solubilization group imparts a high water solubility and low lipophilicity to brigatinib. The colors of the element symbols in the structural formula (**A**) correspond to the colors of the elements in the ball-and-stick representation. Only the hydrogen molecule is shown in black in the structural formula, and in white in the ball-and-stick model.

These results may be explained by medicinal chemistry: brigatinib has structural features that the other two ALK inhibitors do not have, including a dimethylphosphine oxide (DMPO) group [52] (Figure 8A,B).

The DMPO group attached to the C4 aniline substituent is an intramolecular hydrogen bond acceptor. As a result, the activity against ALK was increased by approximately 7-fold as compared to the unsubstituted analog. It also interacts with the GDFG (glycine, aspartic acid, phenylalanine, and glycine) motif and the side chain of L1256, a leucine residing in the adenine binding pocket. Among the other structural features that make brigatinib more specific for ALK is the methoxy group that interacts with the hinge residue, L1198, and the C5 chlorine atom that interacts with the gatekeeper residue, L1196 [52]. These structural features of brigatinib are associated not only with its high affinity for NSCLC cell lines that have acquired resistance to crizotinib or alectinib [25], but also with several pharmacokinetic features such as the high water solubility, low lipophilicity, and low protein-binding capacity of brigatinib, which may contribute to the efficacy of brigatinib in CNS metastases [25,53].

As this analysis is an indirect comparison, it is rather difficult to draw definitive conclusions from the results; however, they may suggest the superior efficacy of brigatinib over alectinib in ALK-p, ALK-inhibitor-naïve, advanced NSCLC with CNS metastasis.

Our ITC does have some limitations. Firstly, the dose of alectinib differs between the two studies of alectinib (ALEX and J-ALEX). However, the target race is different in ALEX and J-ALEX; ALEX is an international collaborative study, whereas J-ALEX is an investigation of Japanese patients. The international dosage approved by the US FDA and the dosage approved in Japan are different due to racial differences. Our analysis suggests that the selected dose of alectinib was appropriate for the different populations in both studies. Therefore, we considered these racial differences while performing our ITC. Secondly, our ITC compared the safety profiles of brigatinib and alectinib for all ALK-p NSCLC patients, but not for ALK-p NSCLC cases with CNS metastasis, because there were no data for this sub-group. However, a comparison of brigatinib and alectinib in patients with CNS metastasis will necessitate the investigation of not only the efficacy, but also the safety profiles. Hence, the evaluation of the safety profile of brigatinib in ALK-p NSCLC patients with CNS metastasis will be an important subject of clinical study. Thirdly, our study indicated that brigatinib was more effective than alectinib in patients with CNS metastases. However, the meta-analysis compared the efficacy of brigatinib and alectinib in all evaluable lesions including CNS metastasis. Therefore, further validation is needed to determine whether our results will be valid even if the target lesion to be evaluated is limited to the CNS metastasis lesion. Finally, this indirect comparison method properly integrates the results of RCTs with certain heterogeneity between studies (although no significant heterogeneity was detected in this study) to compare the drugs. Nevertheless, this approach remains an alternative given the heterogeneity and potential discrepancies between studies. To collect more robust evidence, future large RCTs that directly compare brigatinib and alectinib in ALK-p, ALK-inhibitor-naïve, advanced NSCLC patients are needed.

## 5. Conclusions

In summary, the present ITC was performed according to best practice guidelines to compare the efficacy of brigatinib and alectinib in ALK-p, ALK-inhibitor-naïve NSCLC patients with and without CNS metastasis. Although there were no significant differences between the two drugs, alectinib ranked higher than brigatinib in the overall ALK-p, ALK-inhibitor-naïve NSCLC patient population (with and without CNS metastasis), whereas brigatinib ranked higher than alectinib in the CNS metastasis sub-group. Given that this analysis is an indirect comparison, the results reported here require further validation, such as a direct comparison RCT.

## Figures and Tables

**Table 1:** Key inclusion criteria of the included studies.

Study	Key Inclusion Criteria
ALTA-1L	18 years of age or older
	Locally advanced or metastatic ALK-p NSCLC with at least one measurable lesion
	No previous ALK-targeted therapy
ALEX	18 years of age or older
	Locally advanced or metastatic ALK-p NSCLC with at least one measurable lesion
	Performance status range of 0–2
	No previous systemic treatment for advanced NSCLC
J-ALEX	20 years of age or older
	Stage III B, IV, or postoperative, recurrent, ALK-p NSCLC with at least one measurable lesion
	Performance status range of 0–2
	ALK-inhibitor-naïve Japanese patients with ALK-p NSCLC
	Chemotherapy-naïve or one previous chemotherapy regimen

ALK-p, anaplastic lymphoma kinase (ALK) rearrangement-positive; NSCLC, non-small cell lung cancer.

**Table 2:** Characteristics of studies.

Study	Treatment Arms	*n*	Age (Years): Median (Range)	Female: No. (%)	ECOG PS: No. (%)	Smoking Status: No. (%)	Histological Type: No. (%)	Stage of Disease at Entry: No. (%)	CNS Metastasis: No. (%)
ALTA-1L	Brigatinib 180 mg	137	58 (27–86)	69 (50)	PS0–1: 131 (96)	Never: 84 (61)	Adeno: 126 (92)	III B: 8 (6)	40 (29)
	once daily				PS2: 6 (4)	Former: 49 (36)	Squamous: 4 (3)	IV: 129 (94)	
	(7-day run-in					Current: 4 (3)	Other: 7 (4)		
	period of 90 mg								
	once daily)								
	Crizotinib 250 mg	138	60 (29–89)	81 (59)	PS0–1: 132 (96)	Never: 75 (54)	Adeno: 137 (99)	III B: 12 (9)	41 (30)
	twice daily				PS2: 6 (4)	Former: 56 (41)	Squamous: 0 (0)	IV: 126 (91)	
						Current: 7 (5)	Other: 1 (1)		
		total, 275							
ALEX	Alectinib 600 mg	152	58 (25–88)	84 (55)	PS0–1: 142 (93)	Never: 92 (61)	Adeno: 137 (90)	III B: 4 (3)	64 (42)
	twice daily				PS2: 10 (7)	Former: 48 (32)	Squamous: 5 (3)	IV: 148 (97)	
						Current: 12 (8)	Other: 10 (7)		
	Crizotinib 250 mg	151	54 (18–91)	87 (58)	PS0–1: 141 (93)	Never: 98 (65)	Adeno: 142 (94)	III B: 6 (4)	58 (38)
	twice daily				PS2: 10 (7)	Former: 48 (32)	Squamous: 2 (1)	IV: 145 (96)	
						Current: 5 (3)	Other: 7 (5)		
		total, 303							
J-ALEX	Alectinib 300 mg	103	61.0 (27–85)	62 (60)	PS0–1: 101 (98)	Never: 56 (54)	Adeno: 100 (97)	III B: 3 (3)	16 (16)
	twice daily				PS2: 2 (2)	Former: 45 (44)	Squamous: 2 (2)	IV: 76 (74)	
						Current: 2 (2)	Other: 1 (1)	postoperative	
								recurrence: 24 (23)	
	Crizotinib 250 mg	104	59.5 (25–84)	63 (61)	PS0–1: 102 (98)	Never: 61 (59)	Adeno: 103 (99)	III B: 3 (3)	31 (30)
	twice daily				PS2: 2 (2)	Former: 40 (38)	Squamous: 0 (0)	IV: 75 (72)	
						Current: 3 (3)	Other: 1 (1)	postoperative	
								recurrence: 26 (25)	
		total, 207							

Total *n* = 785 patients; the intention-to-treat (ITT) population included patients who were randomized regardless of whether an intervention was performed. ECOG, Eastern Cooperative Oncology Group; PS, performance status; CNS, central nervous system.
